# Monolithic Silicon‐Photonics Sign‐Sign Dot‐Product Circuits Toward Artificial Intelligence and Multiple‐Input Multiple‐Output Accelerators

**DOI:** 10.1002/nap2.70063

**Published:** 2026-04-19

**Authors:** Sumilak Chaudhury, Karl Johnson, Nikola Alic, Bill Lin, Yeshaiahu Fainman, Tzu‐Chien Hsueh

**Affiliations:** ^1^ University of California, San Diego La Jolla California USA

**Keywords:** dot product, intensity modulation, intensity photodetection, linear algebra, matrix multiplication, micro‐ring resonator, monolithic integration, multiply‐accumulate, silicon photonics, WDM

## Abstract

This research leverages advanced monolithic silicon‐photonics integrated‐circuit manufacturing capabilities to realize system‐on‐chip photonic‐computing‐based linear‐algebra accelerators for a wide range of applications in artificial intelligence, machine learning, and multiple‐input multiple‐output wireless technology. With holistic codesign in both photonic and electronic domains, strategic electrical‐to‐optical signal conversion, a differential intensity‐modulation technique, and a dual rail‐to‐rail photodetection architecture, the monolithic photonic‐electronic test chip of a sign‐sign dot‐product accelerator achieves 8.92‐Gb/s/MAC computation throughput with 2.22‐pJ/b/MAC energy consumption for next‐generation large‐scale linear‐algebra computing hardware targeting higher than one TMAC/s/mm^2^ computation density with only tens of fJ/MAC energy consumption.

## Graphical abstract

This research leverages advanced monolithic silicon‐photonics integrated‐circuit manufacturing technology with strategic electrical‐to‐optical signal conversions, differential intensity‐modulation technique, and dual rail‐to‐rail photodetection architecture to realize an on‐chip photonic‐electronic sign‐sign dot‐product accelerator achieving 8.92‐Gb/s/MAC computation throughput with 2.22‐pJ/b/MAC energy consumption for next‐generation large‐scale linear‐algebra computing hardware targeting higher than one TMAC/s/mm^2^ computation density with only tens of fJ/MAC energy consumption.

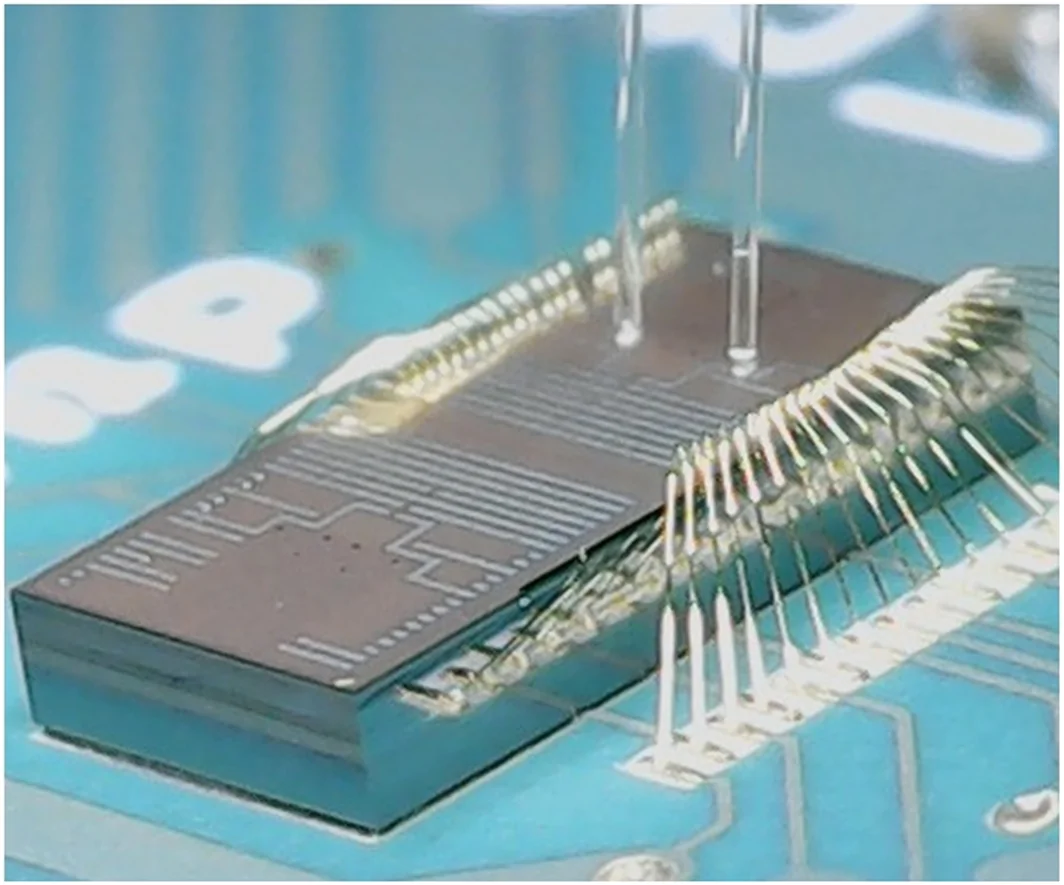

## 1 Introduction

The exponential rise in the complexity of artificial intelligence (AI) and machine learning (ML) models continues to explode rapidly. For example, the next generation of large language models (LLMs) is expected to grow by one order of magnitude or more, with its corresponding computing requirements for training and inference also increasing at a similar rate [1, 2]. These super‐exponential growth trends are likely to continue, as there appear to be no scaling limitations on learning capacity with respect to the underlying LLM architecture [3] or the availability of data from which to learn. Similar explosive growth is occurring in other AI and ML models, particularly in diffusion models for generative AI (GenAI) applications, such as text‐to‐image and text‐to‐video generation [4, 5]. In addition, the number of AI users and their frequency of use create another dimension of an explosion in computing demand for runtime inference [6]. These different dimensions of exponential growth mean that existing digital computing architectures will surely be unable to keep up with the disruptive increases in throughput and energy requirements of the linear‐algebra computations in these AI and ML models.

Meanwhile, the newly available millimeter‐wave (mmWave) spectrum in emerging 5G/6G wireless systems, coupled with advanced massive multiple‐input multiple‐output (MIMO) technology, will enable new wireless applications with robust, low‐latency requirements to support multiuser mobility, including wireless extended reality (XR) experiences for large group‐based applications. Such advanced MIMO‐based wireless XR platforms will need to accommodate multiple users in large groups, allowing them to freely roam through common areas while jointly experiencing interactive, immersive virtual or mixed reality environments. Although such wireless XR experiences have numerous applications in training, education, and entertainment, delivering high‐resolution digital XR experiences requires a tremendous amount of bandwidth and the aggressive use of multiple antennas to achieve the necessary data rates through parallel data streams, thereby substantially increasing the dimension of the matrices involved in the MIMO decoding process. In other words, the linear‐detection‐based MIMO decoding [7] will require the base stations to estimate the channel matrix inversions quickly enough to capture channel condition variations accurately. However, the complexity of the linear‐algebra computation in MIMO channel decoding increases cubically with the number of users that need to be served simultaneously, which again makes the existing digital computing architectures a key limiting factor for the future scaling of massive MIMO systems.

When the advancement of AI and MIMO workloads far outpaces Moore's law [8] and the energy/area bottleneck of classical von Neumann digital computing architectures, silicon‐photonics (SiPh) integrated‐circuit (IC) process technologies and modern optical communication techniques, used for high‐volume data transmissions [9, 10, 11, 12, 13, 14], have widely utilized the inherent parallelism, a high degree of connectivity, and speed‐of‐light propagation of photonics associated with the wavelength‐division‐multiplexing (WDM) technique [15] to perform computation tasks such as passive Fourier transforms [16] and matrix operations [8, 17, 18, 19, 20, 21, 22, 23, 24], exhibiting superior advantages of photonic computing in bandwidth capacity, processing latency, and energy consumption. Recently, the availability of commercial monolithic SiPh semiconductor process technology, such as GlobalFoundries (GF) 45SPCLO [25, 26], has enabled the opportunity to explore holistic codesign approaches that leverage the unique and complementary capabilities of photonics and CMOS electronics to advance the state of computing. That is, monolithically consolidating all required photonic devices and electronic circuits for computing systems on a single silicon substrate can significantly reduce power/area/integration overhead due to heterogeneous interfaces, electrostatic‐discharge (ESD) protection diodes, chip bumps/pads, and interposers/packages among separate photonic and electronic dies.

Therefore, to realize the linear‐algebra computations in the chip‐scale hardware physical layers for super‐exponentially growing computation workloads, as mentioned, photonic computing via monolithic SiPh fabrication and integration [25, 26] is the primary implementation strategy of this article to preliminarily demonstrate a monolithic photonic‐electronic (MPE) sign‐sign dot‐product accelerator achieving 8.92‐Gb/s/MAC computation throughput with 2.22‐pJ/b/MAC energy consumption for next‐generation large‐scale linear‐algebra computing hardware targeting higher than one TMAC/s/mm^2^ computation density with only tens of fJ/MAC energy consumption [27, 28].

The remainder of this article about the MPE dot‐product accelerator is organized as follows: The mathematical expressions and parameter definitions are described in Section 2. The signal conversion strategies are elaborated in Section 3. The circuit algorithm and architecture are described and analyzed in Section 4. The measurement results and characterizations of the preliminary test chip are summarized in Section 5. The performance summary and future work are discussed in Section 6.

## 2 Dot‐Product Computations

Almost all the computing algorithms in ML [27], signal processing in communication systems [28], and convolutional neural networks in neuromorphic computing [8] are converted through machine languages and assembly languages down to the hardware physical layer and then executed by tons of matrix‐vector multiplications (MVMs), which are the key linear‐algebra functionality [29] to form various LLMs, diffusion models, and channel decoding algorithms in AI and MIMO applications.

A simple MVM example, denoting the signal symbols and terminology of this article, is shown in Figure 1, where the 4‐by‐4 matrix Y_4×4_ is the multiplicand, the 4‐by‐1 vector Z_4×1_ is the multiplier, and the 4‐by‐1 vector YZ_4×1_ is the product of this MVM computation. Each element of YZ_4×1_ is the outcome of the dot‐product computation (DPC) [29] between each row vector of Y_4×4_ and the vector Z_4×1_. For instance, the element yz_1_ is the DPC outcome of the first‐row vector of Y_4×4_ and the vector Z_4×1_; that is, the DPC outcome yz_1_ is the accumulated sum of all element‐element multiplications (EEMs) between the elements y_11_, y_12_, y_13_, and y_14_ and the elements z_1_, z_2_, z_3_, and z_4_, as shown in Figure 1a. In the process of forming yz_1_, each EEM followed by an addition operation is equivalent to a “multiply‐accumulate” or “multiply‐add” computation (MAC), for example, “y_11_·z_1_ +,” which has been the most essential computation unit in all digital and general‐purpose processors [8, 27, 28, 30, 31, 32, 33]. In this example, performing each DPC requires 4 MACs, and performing this MVM requires 4 DPCs, which is equivalent to 16 MACs in total. In other words, the bottom‐up computation architecture is established from the building blocks of EEMs, MACs, DPCs, and MVMs to the levels of LLMs, diffusion models, or channel decoding algorithms. Therefore, the essential goal of the MPE accelerator is to perform high‐volume, high‐speed MACs and DPCs with minimal energy consumption.

**FIGURE 1:**
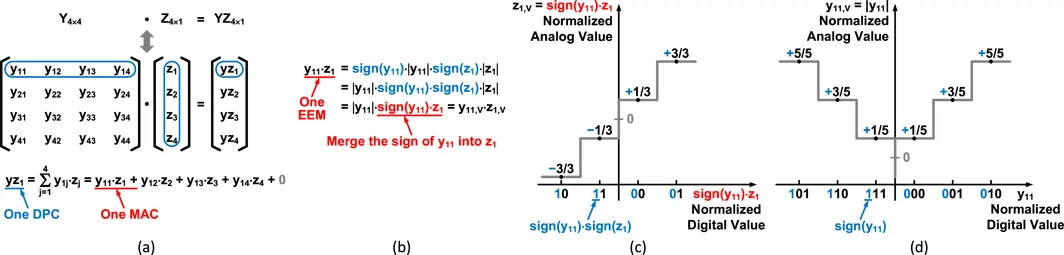
(a) The mathematical expressions of an MVM, a DPC, and a MAC. (b) The equivalent EEM mathematical expression for the hardware implementation. (c) The mid‐rise D/A conversion from digital “sign(y_11_)·z_1_” to analog “z_1,V_.” (d) The mid‐rise D/A conversion from digital “y_11_” to analog “y_11,V_.”

It is also important to note that, specifically in AI and MIMO applications [27, 28], the multiplicand matrix Y and its elements y_*ij*_ are updated slowly according to the assigned computation tasks. At the same time, the multiplier vector Z and its elements z_*j*_ are high‐speed data that need to be processed and computed in real time. Therefore, the computation speed in any MVM implementation is dominated by the signal processing latency in the hardware, from the time the input Z is asserted to the time the output YZ settles, which is similar to the concept of combinational logic delays in a digital circuit implementation.

With the basic definitions above, the MPE accelerator implementation described in this article can be more straightforward in specifying which parts of the integrated‐circuit hardware are used to realize the corresponding operations. Although the MPE accelerator employs the concept of analog signal processing in the photonic domain, that is, photonic computing, its input/output interfaces and operational cycles are still governed by digital circuits, data, and clocks. One of the key features is that the computation latency in the photonic domain of the MPE accelerator is relatively short, allowing the clock frequency per computation to be increased and thereby improving computation throughput compared to existing digital processors. In other words, the electronic portion of the MPE accelerator dominates its overall computing speed. Additionally, because of the workload explosions in today's AI and MIMO applications, a larger MVM dimension that a piece of computing hardware can handle within a specific period of time presents a higher computation throughput associated with the corresponding physical size and energy consumption of the hardware [27, 28], which are further discussed in Section 6.

## 3 Signal Conversion Strategies

The realization of the MPE accelerator requires multiple stages of signal conversions, including from an electrical digital signal to an electrical analog signal (D/A), from an electrical analog signal to an optical carrier power (E/O), from an optical carrier power to an electrical analog signal (O/E), and then from an electrical analog signal to an electrical digital signal (A/D). In addition to these inevitable signal conversion processes, this article incorporates several signal mapping strategies to effectively simplify the MPE accelerator complexity and calibration accuracy requirements while maintaining bandwidth, energy efficiency, and functionality.

Additionally, it is crucial to support the sign‐sign multiplication (SSM) capability, which allows all multiplicands, multipliers, and their products in the operations of EEM, MAC, DPC, and MVM to preserve their informative polarities or sign bits. However, it is not intuitive to maintain the signal polarities when using the optical intensity‐modulation technique in WDM [15] because the nature of the intensity modulation is to encode or map the data information into the optical carrier “power,” which is always a real number larger than or equal to zero, during the E/O conversion process.

### 3.1 Sign‐Bit Preconsolidation

To efficiently realize the SSM using the optical intensity modulation in the MPE accelerator, the first strategy is to merge the sign bit of the multiplicand into the multiplier, as shown in Figure 1b, where y_11_ and z_1_ are signed digital data represented in 1's complement format [34]. Before calculating “y_11_·z_1_,” it is fast and straightforward for digital circuits to merge the sign bit of y_11_ into z_1_ to become a multiplication of a non‐negative datum |y_11_| and sign(y_11_)·z_1_. That is, if sign(y_11_) is positive, all bits of z_1_ stay the same: sign(y_11_)·z_1_ = z_1_; if sign(y_11_) is negative, all bits of z_1_ flip: sign(y_11_)·z_1_ = −z_1_. In this way, the signal polarity processing of this EEM has been taken care of and embedded in “sign(y_11_)·z_1_” even before any signal conversion or computation has begun. From the standpoint of photonic computing, the sign‐bit preconsolidation process offers multiple benefits, including better linearity, higher dynamic range (DR), and lower calibration costs, as revealed throughout this article.

### 3.2 Mid‐Rise D/A Conversions

The second strategy is to use mid‐rise mapping [34] instead of mid‐tread mapping [34] for the D/A conversions. For example, suppose the digital word z_1_ contains four possible values across the normalized DR between −1 and +1; then, all possible values of z_1_ in the digital domain can be represented by two binary bits in 1's complement format. Similarly, all possible values of “sign(y_11_)·z_1_” have the same expressions as shown on the *x*‐axis of Figure 1c, where the most significant bit (MSB) is the sign bit. The mid‐rise D/A conversion linearly maps all possible values of a digital word, for example, sign(y_11_)·z_1_, to their corresponding analog values z_1,V_ without a mapping option to an exact zero, as shown on the *y*‐axis of Figure 1c. For another example, suppose the digital word y_11_ contains six possible values across the normalized DR between −1 and +1; then, all possible values of y_11_ in the digital domain can be represented by three binary bits in 1's complement format, as shown on the *x*‐axis of Figure 1d, where the MSB is the sign bit. In this example, due to the strategy of merging sign(y_11_) into z_1_, as shown in Figure 1b, both positive and negative values of y_11_ are converted to their absolute analog values y_11,V_ = |y_11_| through mid‐rise mapping, as indicated on the *y*‐axis of Figure 1d.

Regardless of the scenario in Figure 1c or Figure 1d, a mid‐rise D/A conversion never generates an outcome at exact zero in the analog domain. Therefore, any value close to or equal to zero is always quantized to the positive or negative least significant bit (LSB) divided by two (i.e., LSB/2 or −LSB/2), which still satisfies the signal conversion and quantization theorems, as well as the computing functionality. In other words, using mid‐rise mapping eliminates the need to generate an electrical analog signal or optical carrier power with zero magnitude. As a result, the E/O circuit/device performance requirements and thermal control calibration overhead can be significantly simplified, as revealed in Section 3.3.

### 3.3 Independently Optimized E/O Dynamic Ranges

The third strategy is to define the independent DRs for the linear E/O conversions of the multiplicand and multiplier based on the properties of constant data values or high‐speed data transition patterns.

The MPE accelerator utilizes the optical‐intensity responses of a photonic microring modulator (MRM) [35] to perform E/O conversions because of its compact silicon area associated with low energy consumption sourced from electronic drivers or digital‐to‐analog converters (DACs) [9, 10, 11]. In the GF45SPCLO SiPh process technology [25, 26], two electrical control knobs can be used to influence the power spectrum transfer function of an MRM (TF_MRM_) from its optical input port to its optical output port (i.e., Through Port): One is the P/N junction reverse bias voltage *V*_NP_, and the other is the thermal control voltage *V*_th_. TF_MRM_ is essentially a periodic notch‐filtering response across a specific span of the optical wavelength spectrum, as shown by the measured TF_MRM_ in Figure 2a,d, where the wavelength periodicity between the notch points is referred to as the free spectral range (FSR) [36] of the MRM. Overall, TF_MRM_ is mainly a function of the wavelength (*λ*), *V*_NP_, and *V*_th_, expressed as TF_MRM_{*λ*, *V*_NP_, *V*_th_}.

**FIGURE 2:**
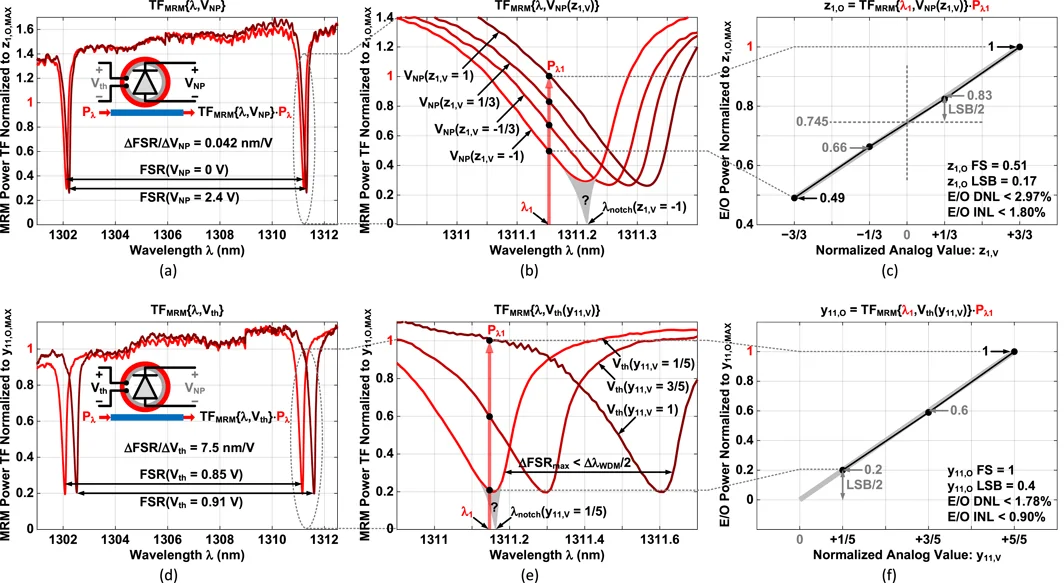
The normalized MRM optical power spectrum TFs showing (a) the FSR shifts under two different *V*_NP_ voltages and (b) the zoomed‐in FSR or notch–response shifts under four different *V*_NP_(z_1,V_) voltages. (c) The high‐speed multiplier static E/O conversion transfer curve. The normalized MRM optical power spectrum TFs showing (d) the FSR shifts under two different *V*_th_ voltages and (e) the zoomed‐in FSR or notch–response shifts under three different *V*_th_(y_11,V_) voltages. (f) The constant multiplicand static E/O conversion transfer curve. Note that the powers used for the z_1,O_ and y_11,O_ normalizations are different; that is, the multiplier and multiplicand have their own independently optimized E/O conversion DRs (as well as LSBs) described in Section 3.3.

The primary effect of influencing TF_MRM_ by changing *V*_NP_ or *V*_th_ is the corresponding FSR shift, which is quantified by the FSR sensitivity, that is, ΔFSR/Δ*V*_NP_ or ΔFSR/Δ*V*_th_. For example, the measured ΔFSR/Δ*V*_NP_ and ΔFSR/Δ*V*_th_ are about 0.042 nm/V in Figure 2a and 7.5 nm/V in Figure 2d, respectively, at a wavelength of around 1311.15 nm. Besides the different resolutions of the FSR sensitivities with respect to Δ*V*_NP_ and Δ*V*_th_, the speeds at which ΔFSR responds to Δ*V*_NP_ and Δ*V*_th_ are also significantly different. For example, the FSR can be altered with *V*_NP_ at rates exceeding 25 GHz [25, 26], which is why MRMs are widely used in high‐speed data communication [12, 13, 14]. In contrast, the FSR can be altered slowly with *V*_th_ roughly in the tens‐of‐kilohertz range, which strongly depends on the heat‐dissipation capability of the on‐chip environment. Therefore, the MPE accelerator utilizes *V*_NP_(z_1,V_) as a function of z_1,V_ for the high‐speed multiplier E/O conversion from z_1,V_ to z_1,O_, while mainly using *V*_th_(y_11,V_) as a function of y_11,V_ for the low‐speed multiplicand E/O conversion from y_11,V_ to y_11,O_.

For the high‐speed multiplier E/O conversion, as shown in Figure 2b, when *V*_th_ is a constant, four different voltages of *V*_NP_(z_1,V_), generated by a high‐speed DAC (HS‐DAC) [27, 28] according to four different values of z_1,V_, create four different FSR shifts so that an *λ*_1_ light wave (or WDM carrier) with a constant optical power *P*_*λ*1_ injection can experience four different corresponding magnitudes of the power transfer function, TF_MRM_{*λ*_1_, *V*_NP_(z_1,V_)}, and then generate four different optical output powers z_1,O_ = TF_MRM_{*λ*_1_, *V*_NP_(z_1,V_)}·*P*_*λ*1_, accordingly. This entire E/O conversion sequence is shown as follows: z_1,V_ → *V*_NP_(z_1,V_) → TF_MRM_{*λ*_1_, *V*_NP_(z_1,V_)} → TF_MRM_{*λ*_1_, *V*_NP_(z_1,V_)}·*P*_*λ*1_ → z_1,O_, and the overall E/O conversion transfer curve from z_1,V_ to z_1,O_ is summarized in Figure 2c. In the time domain, when the high‐speed data z_1,V_ vary among their four possible values at a specific symbol rate, for example, 2 GSym/s (i.e., the reciprocal of a 500‐ps time duration that each data value holds), the MRM output *λ*_1_ carrier power z_1,O_ varies accordingly based on the transfer curve relation shown in Figure 2c, which is generally referred to as the “intensity modulation.”

Multiple aspects are discussed as follows: The first one is the E/O conversion linearity. The primary causes of nonlinearity during this E/O conversion are the circuit nonideality of the DAC [27, 28] and inconsistent FSR sensitivity and quality factor of the MRM [37, 38]. Additionally, both the DAC and MRM nonlinearities contain both static (steady‐state) and dynamic (transient‐state) components. Because the DAC and MRM bandwidths in GF45SPCLO are well above the 2‐GSym/s multiplier z_1_ symbol rate, the dynamic components of the DAC and MRM nonlinearities are negligible. Meanwhile, the aggregate DAC and MRM static differential nonlinearity (DNL) and integral nonlinearity (INL), which reflect the errors of the E/O transfer curve deviating from the ideal straight gray line shown in Figure 2c, are less than 2.97% (= 1 LSB of 5.07 bits) and 1.80% (= 1 LSB of 5.79 bits), respectively, to ensure that the accuracy of each MAC and DPC outcome is better than the targeted specification, for example, 4 bits.

The second one is the E/O conversion DR, which is intentionally designed to be relatively small, covering only 51% (from 0.49 to 1), for example, of the normalized full‐scale optical power, z_1,O,MAX_, as shown in Figure 2c. A smaller DR, confining the maximum, minimum, and all possible TF_MRM_{*λ*_1_, *V*_NP_(z_1,V_)}, that is, the black dots in Figure 2b, to being distributed in a smaller portion of the monotonic negative‐slope profile of TF_MRM_, can bring multiple advantages: (i) A smaller DR can improve the E/O conversion linearity due to minor slope variations across all possible TF_MRM_{*λ*_1_, *V*_NP_(z_1,V_)}. Note that although a smaller DR makes the signal‐to‐noise ratio lower, the noise issue (i.e., the noise floor in the spectrum) is much less severe than the nonlinearity issue (i.e., harmonics in the spectrum) in affecting the accuracy (i.e., the effective number of bits, ENOB) of this fully on‐chip analog computing. (ii) A smaller DR requires a lower amount of energy consumption sourced from the DAC, which is proportional to the delta between the maximum *V*_NP_(z_1,V_ = 1) = 2.4 V and the minimum *V*_NP_(z_1,V_ = −1) = 0 V. (iii) Regardless of the linearity, a correct E/O conversion has at least to be monotonic, which requires *λ*_1_ always to stay lower than *λ*_notch_(z_1,V_ = −1), as shown in Figure 2b, biased by the static thermal control voltage *V*_th_. Otherwise, any *λ*_notch_(z_1,V_) lower than *λ*_1_ can cause *P*_*λ*1_ to experience both the negative and positive slopes of TF_MRM_ across all possible values of z_1,V_ and result in a nonmonotonic and malfunctioned E/O conversion. A large DR would need to bias *λ*_1_ as close as possible to *λ*_notch_(z_1,V_ = −1) for accommodating the full DR in the negative slope of TF_MRM_, which requires a dedicated thermal control loop for *V*_th_ to accurately preserve the relation of *λ*_1_ < *λ*_notch_(z_1,V_ = −1) with a tiny margin between *λ*_1_ and *λ*_notch_(z_1,V_ = −1). On the other hand, a small DR can allow *λ*_1_ well below *λ*_notch_(z_1,V_ = −1) while maintaining the E/O monotonicity without sacrificing too much of the E/O linearity so that the area/power overhead for the thermal control loop can be minimized. (iv) As the cone‐shaped gray area shown in Figure 2b, the MRM quality factor determines how deep the notch point can be achieved, which can also be quantified by the MRM extinction ratio (ER). In reality, this cone‐shaped gray area is full of uncertainty in terms of its characteristics because the sharp and narrow notch behavior causes high sensitivity to process variation and on‐chip environmental temperature of the MRM; even under well‐controlled thermal conditions, it requires an extremely high‐resolution tunable laser source to capture the exact values of *λ*_notch_. In short, a small DR can perform the E/O conversion well without the knowledge of the cone‐shaped gray area or an excellent ER from the MRM.

The third one, more importantly, is a dual‐polar analog DR converting to a unipolar optical power DR, as shown in Figure 2c. In other words, after the E/O conversion, the full DR of z_1,V_ from −1 to +1 is inevitably shifted to a completely positive DR of z_1,O_ from 0.49 to 1, for example, which offers one advantage and one issue: (i) The advantage is that the ideal E/O DR and linearity are self‐defined by the straight line between the minimum and maximum of z_1,O_, that is, 0.49 and 1, respectively. Therefore, the equivalent zero value of z_1,V_ is automatically mapped to the midpoint or average value between the minimum and maximum of z_1,O_, that is, 0.745 in this example. This is known as self‐referenced (or differential) signaling [39], where the signal quantity is automatically balanced at the midpoint of the entire DR, resulting in zero “signal offset” during the conversion process. Note that although there is never an exact zero value of z_1,V_ needing to be converted to the midpoint of the z_1,O_ DR due to the mid‐rise mapping discussed in Section 3.2, the equivalent zero “signal offset” in the z_1,O_ DR is helpful to reduce the INL and DNL because of no errors at the minimum, middle, and maximum values across the DR of z_1,O_. Overall, this self‐referenced E/O conversion has the advantage of zero “signal offset,” and the LSB of z_1,O_ is directly defined by the “delta” between adjacent TF_MRM_{*λ*_1_, *V*_NP_(z_1,V_)} values. (ii) The issue is that the E/O conversion through the optical intensity modulation inevitably shifts or biases the data information to the median value of the z_1,O_ DR, for example, 0.745, as mentioned, which is an optical DC power bias not representing any information in the original data z_1_. Additionally, during the DPC process, for example, y_11,O_·z_1,O_ + y_12,O_·z_2,O_, in the photonic domain, the 0.745 median power of z_1,O_ is scaled by y_11,O_ and then added to the median power of y_12,O_·z_2,O_ to form a y_11_‐ and y_12_‐dependent optical DC power bias, which again is not related to the original data z_1_, z_2_, y_11_, and y_12_ and their DPC outcome. In short, to perform the SSM correctly in each DPC, the inevitable data‐dependent optical DC power bias must be properly removed without affecting the targeted computation outcome. The proposed differential intensity‐modulation technique in the photonic domain with a dual rail‐to‐rail photodetection architecture, discussed in Section 4, can effectively resolve this issue.

For the low‐speed multiplicand E/O conversion, as shown in Figure 2e, when *V*_NP_ is a constant, three different voltages of *V*_th_(y_11,V_), generated by a low‐power DAC (LP‐DAC) [27, 28] according to three different values of y_11,V_, create three different FSR shifts so that the *λ*_1_ WDM carrier with a certain optical power *P*_*λ*1_ injection can experience three different corresponding amounts of the power transfer function magnitude, TF_MRM_{*λ*_1_, *V*_th_(y_11,V_)}, and then generate three different optical output powers y_11,O_ = TF_MRM_{*λ*_1_, *V*_th_(y_11,V_)}·*P*_*λ*1_, accordingly. This entire E/O conversion sequence is shown as follows: y_11,V_ → *V*_th_(y_11,V_) → TF_MRM_{*λ*_1_, *V*_th_(y_11,V_)} → TF_MRM_{*λ*_1_, *V*_th_(y_11,V_)}·*P*_*λ*1_ → y_11,O_, and the overall E/O conversion transfer curve from y_11,V_ to y_11,O_ is summarized in Figure 2f.

Conceptually, the E/O conversions of the multiplicand and multiplier are similar; however, a few distinct properties require clarification. (i) The modulation bandwidths of *V*_NP_ and *V*_th_ are significantly different, as mentioned earlier in Section 3.3. Therefore, *V*_NP_(z_1,V_) is primarily used for the high‐speed multiplier E/O conversion with a constant *V*_th_ for static E/O DR tuning. In contrast, *V*_th_(y_11,V_) is used mainly for the constant multiplicand E/O conversion with a constant *V*_NP_ for static E/O linearity tuning. (ii) *P*_*λ*1_ must always experience the negative slopes of TF_MRM_ across all possible y_11,V_ to result in a monotonic E/O conversion, as shown in Figure 2e,f. (iii) More importantly, the unipolar analog DR of y_11,V_ is converted to the unipolar optical power DR of y_11,O_. In other words, the ideal E/O DR and linearity are defined by the straight line between the exact zero and the maximum y_11,O_, as shown in Figure 2f. Although both y_11,V_ and y_11,O_ never have an exact zero value due to the mid‐rise mapping mentioned in Section 3.2, the LSB of y_11,O_ is determined by how small TF_MRM_{*λ*_1_, *V*_th_(y_11,V_ = 1/5)} can be to form the delta between the exact zero and LSB/2 of y_11,O_. To avoid TF_MRM_{*λ*_1_, *V*_th_(y_11,V_ = 1/5)} falling into the cone‐shaped gray area, as shown in Figure 2e, with high performance uncertainty, *λ*_notch_(y_11,V_ = 1/5) is chosen by a proper *V*_th_(y_11,V_ = 1/5), as shown in Figure 2e, which determines the minimum y_11,O_ = TF_MRM_{*λ*_1_, *V*_th_(y_11,V_ = 1/5)}·*P*_*λ*1_ = LSB/2 of y_11,O_. Then, the rest of the y_11,O_ values, including y_11,O,MAX_ = TF_MRM_{*λ*_1_, *V*_th_(y_11,V_ = 1)}·*P*_*λ*1_ = the DR of y_11,O_, are determined accordingly. Therefore, *V*_th_(y_11,V_ = 1) can be decided to shift the FSR and meet the required TF_MRM_{*λ*_1_, *V*_th_(y_11,V_ = 1)}, as shown in Figure 2e. The very nonlinear relation of all *V*_th_(y_11,V_) values can result in a very linear relation of all y_11,O_ values, which is completely feasible because *V*_th_(y_11,V_) and y_11,O_ are essentially constant during the computation process, and only the static linearity of y_11,O_ matters. (iv) The side effect of a large ΔFSR for a large TF_MRM_{*λ*_1_, *V*_th_(y_11,V_ = 1)} is that the whole TF_MRM_{λ, *V*_th_(y_11,V_ = 1)} can start affecting the adjacent WDM carrier located at (*λ*_1_ ± Δλ_WDM_), that is, channel‐to‐channel cross talk. The simulation result shows that ΔFSR_max_ must be less than Δλ_WDM_/2 = 0.5 nm so that the cross talk effect can be confined within ± LSB/2 of the targeted minimum 4‐bit computation accuracy per MAC.

In sum, the high‐speed multiplier E/O conversion is self‐referenced dual‐polar mapping with a high‐resolution LSB (i.e., a smaller step size) and high linearity due to a small DR. The low‐speed multiplicand E/O conversion is zero‐referenced unipolar mapping with a low‐resolution LSB (i.e., a larger step size), a large static DR, and high linearity due to both static *V*_th_ and *V*_NP_ tuning. Additionally, the multiplier and multiplicand can be independently scaled or normalized by z_1,O,MAX_ and y_11,O,MAX_, respectively, without affecting the MAC and DPC outcomes eventually.

## 4 MPE DPC Accelerator Implementation

The primary issue with using optical intensity modulation to perform SSMs is the data‐dependent optical DC median power embedded in the DPC outcomes. This section presents a novel MPE accelerator implementation that incorporates the signal conversion strategies discussed in Section 3 to address this issue while accounting for practical considerations related to device mismatches.

### 4.1 Data‐Dependent Optical Median Power

Based on the signal conversion strategies elaborated in Section 3, the high‐speed multipliers z_1,O_ and z_2,O_ shall independently exhibit one of the four normalized optical power levels, for example, 0.49, 0.66, 0.83, or 1, within each data symbol period, for example, 500 ps. If the data patterns of the high‐speed multipliers are random enough such that all four possible optical power levels have equal probability across the entire computation process, the mean and median values of z_1,O_ or z_2,O_ are equal, for example, 0.745. On the contrary, if the four possible optical power levels do not have equal probability, then the mean values of z_1,O_ or z_2,O_ can vary, but their median values stay at 0.745 regardless of the data pattern distributions. In any case, the focus here is on the median values, not the mean values.

After the EEMs in the photonic domain, all four optical power levels of z_1,O_ and z_2,O_ are scaled by the constant multiplicands y_11,O_ and y_12,O_, respectively, which is exactly what the EEMs shall perform; meanwhile, the median values of z_1,O_ and z_2,O_ are also scaled by y_11,O_ and y_12,O_, respectively, which equivalently shifts the data constellations of the EEM outcomes, y_11,O_·z_1,O_ and y_12,O_·z_2,O_, to center at different median values. For the example shown in Figure 3a, because y_11,O_ = 1, the one‐dimensional photonic data constellation of y_11,O_·z_1,O_ stays the same as z_1,O_, including the four optical power levels, the delta between the adjacent power levels (i.e., LSB = 1 × 0.17 = 0.17), and the median value (i.e., 1 × 0.745 = 0.745). On the other hand, because y_12,O_ = 0.6, the one‐dimensional data constellation of y_12,O_·z_2,O_ is a scaled version of z_2,O_ by 0.6, including the four optical power levels, the delta between the adjacent power levels (i.e., LSB = 0.6 × 0.17 = 0.102), and the median value (i.e., 0.6 × 0.745 = 0.447). Again, the LSB scaled by 0.6 is exactly what the EEM shall perform, but the median value, also scaled by 0.6, may cause an issue. Once the DPC, y_11,O_·z_1,O_ + y_12,O_·z_2,O_, in the photonic domain has been completed, its photonic data constellation is formed by the linear summations of all possible optical power levels of y_11,O_·z_1,O_ and y_12,O_·z_2,O_, as shown in the third column of Figure 3a, where the median value of the photonic DPC outcomes is also the linear summation of the median values of y_11,O_·z_1,O_ and y_12,O_·z_2,O_ (i.e., y_11_ × 0.745 + y_12_ × 0.745 = 1 × 0.745 + 0.6 × 0.745 = 1.192).

**FIGURE 3:**
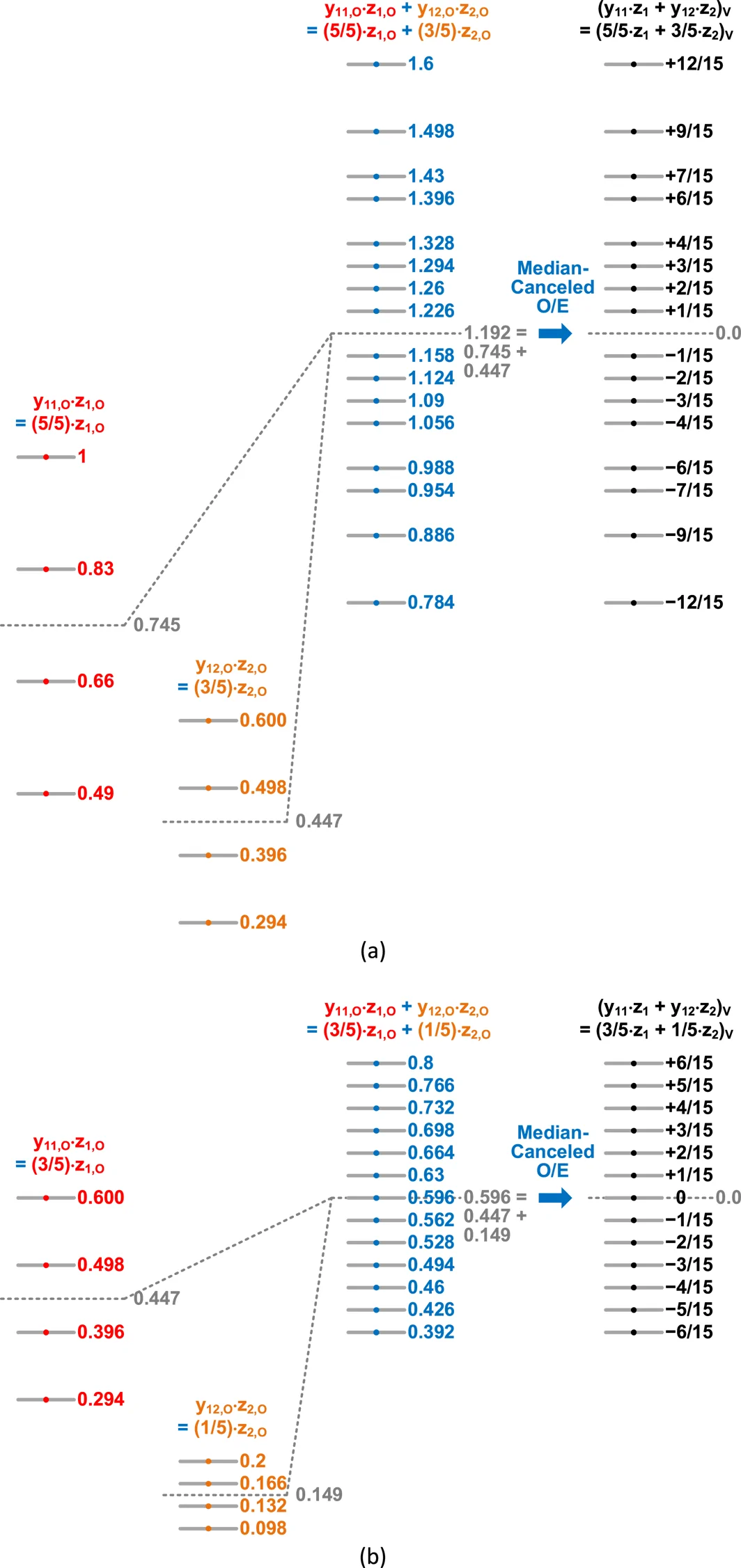
The (a) first and (b) second examples of the ideal data constellations of photonic EEMs (y_11,O_·z_1,O_ and y_12,O_·z_2,O_), photonic DPCs (y_11,O_·z_1,O_ + y_12,O_·z_2,O_), and electronic DPCs (y_11_·z_1_ + y_12_·z_2_)_V_.

Suppose the maximum power value, for example, 1.6, of these photonic DPC outcomes is still within the linear DR of the photodetector (PD) for the O/E conversion, and the photonic DPC data constellation can be preserved except for removing the median value during the O/E conversion and mapping it to an equivalent zero electronic value. In that case, the normalized electronic DPC data constellation shall behave as shown the fourth column of Figure 3a, which is the targeted SSM outcomes of this DPC process. Another example, with y_11,O_ = 0.6 and y_12,O_ = 0.2, is shown in Figure 3b, where it verifies that the median value of the photonic DPC data constellation is data‐dependent, that is, a function of all multiplicands (i.e., y_11_, y_12_, …).

In sum, using optical intensity modulation and detection can successfully perform the SSMs, EEMs, and MACs, and then achieve the final DPC outcomes in the photonic domain in terms of all possible optical power relations in the DPC data constellation, except for the data‐dependent median values, which can be removed by the novel MPE accelerator implementation elaborated in the following subsection.

### 4.2 MPE Sign‐Sign DPC Accelerator Circuit

The block diagram of the MPE dot‐product accelerator circuit is illustrated in Figure 4a. This circuit can perform high‐speed and low‐power DPC with the SSM capability via WDM‐based photonic computing and data‐dependent median‐power cancellation O/E conversions.

**FIGURE 4:**
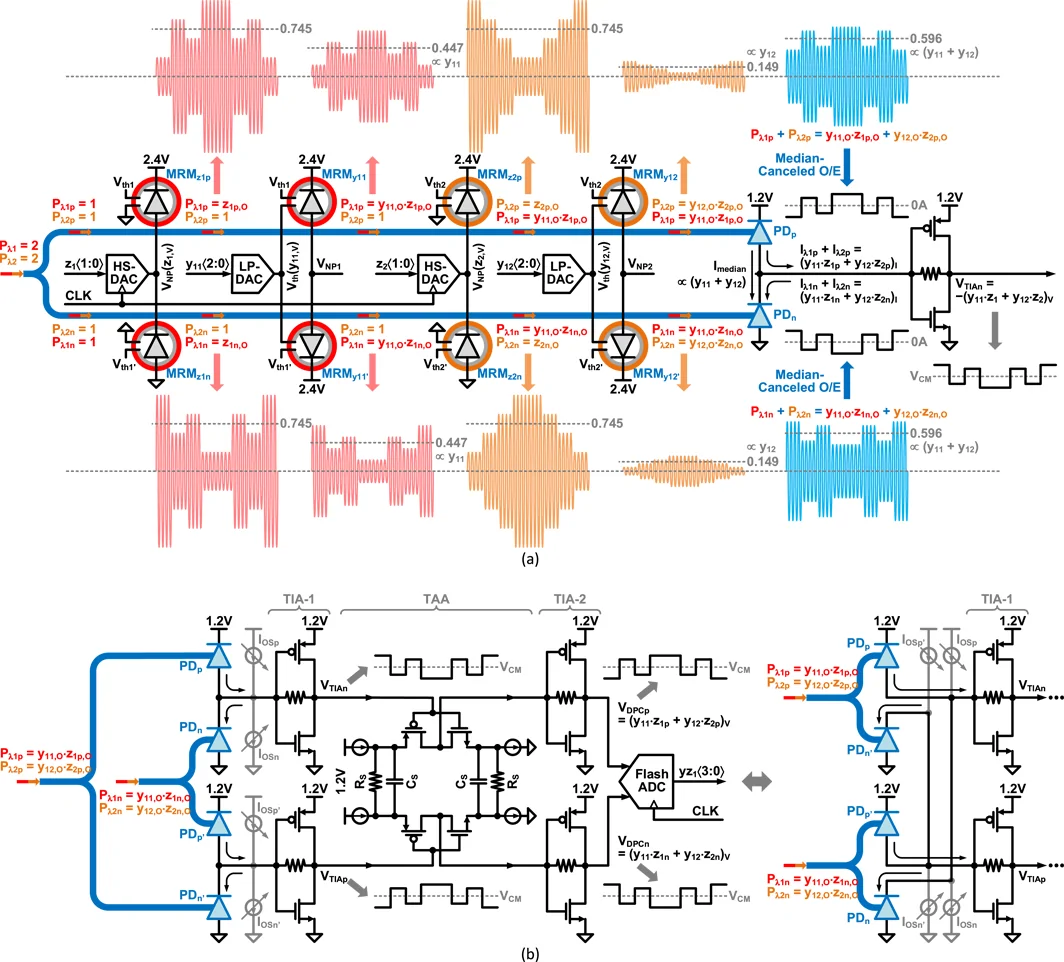
(a) The block diagram of the MPE sign‐sign dot‐product accelerator circuit with the differential intensity‐modulation technique and corresponding time‐domain conceptual light waves. (b) The extension of (a) with dual rail‐to‐rail series PDs to generate differential DPC outcomes in the electronic domain, followed by the broadband fully differential AFE circuit (i.e., TIA‐1 + TAA + TIA‐2) for conditioning the following A/D conversion process. The right side of (b) shows the practical layout floorplan of the interface between the dual rail‐to‐rail series PDs and TIA‐1.

First of all, the signal processing flow of the WDM‐based DPC [27, 28] is illustrated in the upper half of Figure 4a, where the equalized and normalized WDM carrier powers, *P*_*λ*1p_ = *P*_*λ*2p_ = 1, are injected from an external frequency comb source with a WDM channel spacing Δ*λ*_WDM_ = 1 nm between the adjacent carrier wavelengths, *λ*_1_ and *λ*_2_. The resonant wavelengths (*λ*_notch_) of MRM_z1p_ and MRM_y11_ are biased to the proximity of *λ*_1_, as shown in Figure 2b,e, respectively, through the thermal control voltage *V*_th1_. Then, the high‐speed digital multiplier z_1_<1:0> is D/A and E/O converted into z_1p,O_ through an HS‐DAC and MRM_z1p_ performing high‐speed intensity modulation on *P*_*λ*1p_ = 1, as illustrated in Figure 2b, without affecting *P*_*λ*2p_ = 1, so *P*_*λ*1p_ at the MRM_z1p_ output port becomes *P*_*λ*1p_ = TF_MRM_{*λ*_1_, *V*_NP_(z_1,V_)}·1 = z_1p,O_, as the time‐domain conceptual light wave plotted in the top row of Figure 4a, where all four intensity amplitudes (or power levels) of z_1p,O_ are positive, for example, 0.49, 0.66, 0.83, and 1, with a median intensity amplitude of 0.745, and each power level holds for one symbol period, for example, 500 ps, whose symbol‐to‐symbol transitions are governed by the high‐speed clock, CLK. Meanwhile, the constant digital multiplicand y_11_<2:0> is D/A and E/O converted into y_11,O_ = 0.6 in this example through an LP‐DAC and MRM_y11_ performing constant intensity modulation on top of *P*_*λ*1p_ = z_1p,O_, as illustrated in Figure 2e, without affecting *P*_*λ*2p_ = 1, so *P*_*λ*1p_ at the MRM_y11_ output port becomes *P*_*λ*1p_ = TF_MRM_{*λ*_1_, *V*_th_(y_11,V_)}·z_1p,O_ = y_11,O_·z_1p,O_ = 0.6·z_1p,O_, as the time‐domain conceptual light wave plotted in the top row of Figure 4a as well, where the whole envelope of the light wave z_1p,O_ is scaled or shrunk by y_11,O_, including the y_11_‐dependent median intensity amplitude of 0.6 × 0.745 = 0.447 in this example.

Similarly, the resonant wavelengths (*λ*_notch_) of MRM_z2p_ and MRM_y12_ are biased to the proximity of *λ*_2_, as shown in Figure 2b,e, respectively, through the thermal control voltage *V*_th2_. Then, the high‐speed digital multiplier z_2_<1:0> and the constant digital multiplicand y_12_<2:0> are D/A and E/O converted into the z_2p,O_ and y_12,O_ through an HS‐DAC, an LP‐DAC, MRM_z2p_, and MRM_y12_ performing both high‐speed and constant intensity modulations on *P*_*λ*2_ to become z_2p,O_ and then y_12,O_·z_2p,O_ = 0.2·z_2p,O_, as the time‐domain conceptual light waves plotted at the output ports of MRM_z2p_ and MRM_y12_, respectively, in the top row of Figure 4a, where the whole envelope of the light wave z_2p,O_ is scaled or shrunk by y_12,O_ = 0.2, including the y_12_‐dependent median intensity amplitude of 0.2 × 0.745 = 0.149 in this example. Once both *P*_*λ*1p_ = y_11,O_·z_1p,O_ and *P*_*λ*2p_ = y_12,O_·z_2p,O_ are intensity detected by PD_p_, the equivalent DPC outcome, y_11,O_·z_1p,O_ + y_12,O_·z_2p,O_, is the linear intensity‐amplitude (or power‐level) summation of y_11,O_·z_1p,O_ and y_12,O_·z_2p,O_, as the time‐domain conceptual light wave plotted in the top right of Figure 4a, including the y_11_‐ and y_12_‐dependent median intensity amplitude of 0.6 × 0.745 + 0.2 × 0.745 = 0.447 + 0.149 = 0.596 in this example. At this point, if the intensity detection of PD_p_ is well confined within its linear DR region, the PD_p_ output photocurrent shall be a linearly O/E converted electrical quantity directly mapped from (y_11,O_·z_1p,O_ + y_12,O_·z_2p,O_) through the PD_p_ responsivity in A/W. In other words, the data constellation of the PD_p_ output photocurrent (*I*_*λ*1p_ + *I*_*λ*2p_ + *I*_median_) shall exhibit the same characteristics as that of (y_11,O_·z_1p,O_ + y_12,O_·z_2p,O_) shown in the third column of Figure 3b, including the DPC outcomes (*I*_*λ*1p_ + *I*_*λ*2p_) as well as the data‐dependent median value (*I*_median_) but in the electronic domain.

To preserve the DPC outcome and remove the data‐dependent median value, the differential intensity‐modulation technique is proposed in this article and illustrated in the complete picture of Figure 4a, whose bottom‐half photonic circuit is the full replica of the upper half, except that the P/N junction connections of MRM_z1n_, MRM_z2n_, and PD_n_ are flipped and referenced to the low supply (i.e., ground) compared to MRM_z1p_, MRM_z2p_, and PD_p_, respectively. Therefore, the time‐domain conceptual light wave of z_1n,O_, as shown in the bottom row of Figure 4a, exhibits a complementary intensity‐amplitude envelope with respect to that of z_1p,O_, whereas, more importantly, both z_1p,O_ and z_1n,O_ possess an identical median intensity amplitude of 0.745 because, basically, MRM_z1p_ and MRM_z1n_ are biased by the same thermal control *V*_th1_ and driven by the same data z_1_<1:0> and its corresponding analog voltage *V*_NP_(z_1,V_) with symmetrically opposite P/N junction reverse biases. In other words, ideally, both MRM_z1p_ and MRM_z1n_ perform the identical intensity‐modulation process on their own inputs *P*_*λ*1p_ = 1 and *P*_*λ*1n_ = 1, as shown in Figure 2b, but under exactly opposite FSR shifts with respect to a common median value at any moment during the WDM‐based DPC process.

Then, the differentially intensity‐modulated *P*_*λ*1p_ = z_1p,O_ and *P*_*λ*1n_ = z_1n,O_ are both scaled by y_11,O_ = 0.6, in this example, through MRM_y11_ and MRM_y11′_, respectively. Because MRM_y11_ and MRM_y11′_ have completely identical circuit configurations and bias/control voltages, including the thermal control *V*_th1_ = *V*_th1′_, fine‐tuned *V*_NP1_, and constant digital data y_11_<2:0> as well as its corresponding analog voltage *V*_th_(y_11,V_), both MRM_y11_ and MRM_y11′_ perform identical intensity‐modulation processes on their own inputs *P*_*λ*1p_ = z_1p,O_ and *P*_*λ*1n_ = z_1n,O_, as shown in Figure 2e, to become the differential photonic EEM, y_11,O_·z_1p,O_ = 0.6·z_1p,O_ and y_11,O_·z_1n,O_ = 0.6·z_1n,O_, respectively. Therefore, the time‐domain conceptual intensity‐amplitude envelopes of y_11,O_·z_1p,O_ and y_11,O_·z_1n,O_ maintain the same complementary relation as z_1p,O_ and z_1n,O_ and possess a common y_11_‐dependent median intensity amplitude of 0.6 × 0.745 = 0.447, as shown in Figure 4a.

Similarly, the data information carried by *λ*_2_ goes through exactly the same differential intensity‐modulation and intensity scaling processes to generate the differential photonic EEM, y_12,O_·z_2p,O_ = 0.2·z_2p,O_ and y_12,O_·z_2n,O_ = 0.2·z_2n,O_, in this example, with a common y_12_‐dependent median intensity amplitude of 0.2 × 0.745 = 0.149, as shown in Figure 4a. Eventually, the differential photonic DPC is formed by the linear intensity‐amplitude summations, *P*_*λ*1p_ + *P*_*λ*2p_ = (y_11,O_·z_1p,O_ + y_12,O_·z_2p,O_) and *P*_*λ*1n_ + *P*_*λ*2n_ = (y_11,O_·z_1n,O_ + y_12,O_·z_2n,O_), which possess complementary intensity‐amplitude envelopes and an identical y_11_‐ and y_12_‐dependent median intensity amplitude of 0.6 × 0.745 + 0.2 × 0.745 = 0.447 + 0.149 = 0.596, as the time‐domain conceptual light waves plotted in Figure 4a. Once (*P*_*λ*1p_ + *P*_*λ*2p_) and (*P*_*λ*1n_ + *P*_*λ*2n_) are individually intensity detected by PD_p_ and PD_n_ and O/E converted into their own photocurrents, (*I*_*λ*1p_ + *I*_*λ*2p_ + *I*_median_) and (*I*_*λ*1n_ + *I*_*λ*2n_ + *I*_median_), the common current, *I*_median_, shall ideally flow from the 2.4‐V supply to the ground through the matched PD_p_ and PD_n_ to force the differential current, (*I*_*λ*1p_ + *I*_*λ*2p_) − (*I*_*λ*1n_ + *I*_*λ*2n_), to flow out of the common node between PD_p_ and PD_n_ because of Kirchhoff’s current law (KCL). Equivalently, the data‐dependent median value generated during the photonic DPC process is removed by this rail‐to‐rail series PD architecture [40] associated with the proposed differential intensity‐modulation technique, regardless of the values of all the multiplicands, y_11_, y_12_, …, etc. Only the differential DPC information is preserved starting from the moment of generating the differential photocurrent, (*I*_*λ*1p_ + *I*_*λ*2p_) − (*I*_*λ*1n_ + *I*_*λ*2n_) = 2·(*I*_*λ*1p_ + *I*_*λ*2p_), which can be further converted into an electrical voltage quantity, (y_11_·z_1_ + y_1_·z_2_)_V_, whose data constellation finally satisfies the targeted sign‐sign DPC outcome shown in the fourth column of Figure 3b.

Note that the time‐domain electronic single‐ended voltage waveforms in Figure 4 (i.e., *V*_TIAp_, *V*_TIAn_, *V*_DPCp_, and *V*_DPCn_) always contain a constant voltage *V*_CM_, which is a data‐independent DC bias essential for the CMOS transistors to function correctly. From the electronic signal, data, and circuit standpoints, this *V*_CM_ represents an AC ground equivalent to the zero value between the positive and negative data levels, as shown in the fourth column of Figure 3a,b, and it has nothing to do with the median values generated during the WDM‐based photonic computing process that need to be canceled. In any case, before being A/D converted back to a digital word, the analog DPC outcome in the electronic domain is preferably obtained from the difference between its two single‐ended and symmetrical versions with respect to *V*_CM_ (e.g., *V*_TIAp_ − *V*_TIAn_ or *V*_DPCp_ − *V*_DPCn_ in Figure 4) because of the self‐reference advantage and common‐mode noise suppression offered by differential signaling [39]. In the sign‐sign DPC process, as shown toward the right end of Figure 4a, the proposed differential intensity‐modulation technique with the rail‐to‐rail series PD structure can successfully remove the data‐dependent median values, but it produces only a single‐ended DPC outcome in the electronic domain, as indicated by *V*_TIAn_ shown in Figure 4a.

The differential electronic DPC signal can be generated by adding another pair of series PDs, that is, PD_p′_ and PD_n′_, to detect the opposite polarity of the differential photonic DPC signal, as shown in Figure 4b. Specifically, if detecting (*P*_*λ*1p_ + *P*_*λ*2p_) by PD_p_ and (*P*_*λ*1n_ + *P*_*λ*2n_) by PD_n_ can generate one polarity of a differential electronic DPC signal, then detecting (*P*_*λ*1n_ + *P*_*λ*2n_) by PD_p′_ and (*P*_*λ*1p_ + *P*_*λ*2p_) by PD_n′_ can reasonably generate the other polarity of the differential electronic DPC signal. Having both PD_p_ and PD_n′_ receive (*P*_*λ*1p_ + *P*_*λ*2p_) can be achieved by a photonic power splitter, equally dividing (*P*_*λ*1p_ + *P*_*λ*2p_) into PD_p_ and PD_n′_, as shown on the left of Figure 4b; similarly, (*P*_*λ*1n_ + *P*_*λ*2n_) can be equally divided into PD_p′_ and PD_n_. Note that although the photonic power splitters appear to attenuate each photonic DPC signal energy by 3 dB in the photonic domain, the total DPC signal energy is still preserved in two opposite‐polarity single‐ended signals; once the following differential flash ADC circuit [27, 28] takes the difference between the two single‐ended signals, the total signal energy is consolidated again in the electronic domain. In sum, the differential intensity‐modulation technique with two photonic power splitters and two pairs of rail‐to‐rail series PDs can achieve differential sign‐sign (zero‐balanced) DPC outcomes in the electronic domain, including the features of photonic median‐value removal and differential electronic signal generation. As shown by the simulated DPC data constellation, that is, the (*V*_DPCp_ − *V*_DPCn_) eye diagram [39, 41], in Figure 5a using the GF45SPCLO process design kit (PDK) with y_11,O_ = 0.6 and y_12,O_ = 0.2, the proposed MPE sign‐sign DPC accelerator circuit can achieve DNL < 0.63% of DR, INL < 1.00% of DR, and residual median (nonzero median) = −0.33% of DR at the computation throughput of 10 Gb/s/DPC (= 5 bits/Sym/DPC × 2 GSym/s), which matches well with the ideal case illustrated in the fourth column of Figure 3b.

**FIGURE 5:**
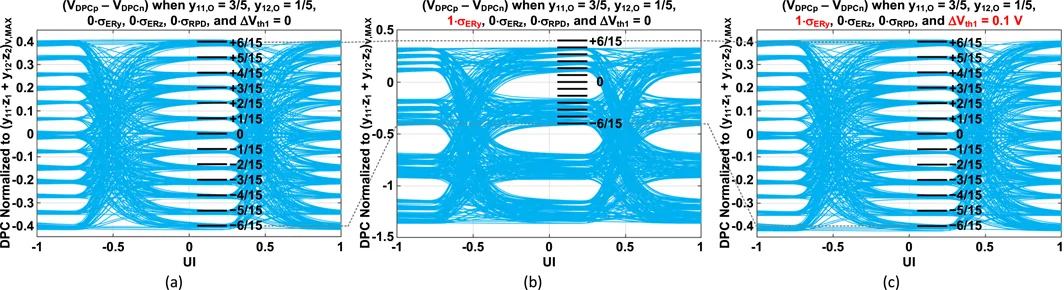
The DPC data constellation (or eye diagram) in the electronic domain (*V*_DPCp_ − *V*_DPCn_) of the MPE sign‐sign DPC accelerator, when z_1_ and z_2_ are high‐speed 2‐bit data running at 2 GSym/s, constant y_11_ = 0.6, and constant y_12_ = 0.2, under (a) the zero‐device‐mismatch condition, (b) one standard‐deviation ER mismatch within the MRM_y11_/MRM_y11′_ pair without any correction mechanism, and (c) one standard‐deviation ER mismatch within the MRM_y11_/MRM_y11′_ pair with the thermal offset tuning mechanism. One UI = one unit interval = one data symbol period = 500 ps.

Additionally, if the physical device placements and signal routings of the photonic waveguides, PDs, and electronic CMOS transistors in GF45SPCLO follow the relations shown on the left of Figure 4b, they would cause undesirable two‐layer waveguide crossovers and very asymmetrical layout floorplans of the MPE accelerator circuit in the photonic domain with the differential intensity‐modulation techniques. This issue can be resolved by pushing the signal crossovers to the electronic domain [27, 28], with the compact signal‐routing flexibility of more than seven‐layer metal/via on‐chip backend resources, as shown on the right of Figure 4b, where the waveguides carrying (*P*_*λ*1p_ + *P*_*λ*2p_) and (*P*_*λ*1n_ + *P*_*λ*2n_) can be completely isolated from each other and maintain a well‐matched and symmetrical floorplan in the photonic domain, as shown in Figure 4a.

Finally, the analog front‐end (AFE) circuit between the PDs and the flash ADC is formed by three stages of differential broadband amplifiers, including the first trans‐impedance amplifier (TIA‐1), the trans‐admittance amplifier (TAA), and the second trans‐impedance amplifier (TIA‐2), as shown in Figure 4b. Each TIA is a current‐to‐voltage amplifier whose feedforward path is formed by complementary P‐type and N‐type transconductance stages, sharing a single DC bias current path from the 1.2‐V supply to the ground, whereas the feedback path of each TIA is a voltage‐to‐current feedback architecture formed by a tunable resistor, which plays a key role in setting the TIA gain, bandwidth, and input/output impedance across all different process, voltage, and temperature (PVT) conditions [41, 42]. In short, TIA‐1 first converts the differential photocurrent from the PD_p_/PD_n_ and PD_p′_/PD_n′_ pairs to the differential voltage of the V_TIAp_/V_TIAn_ pair. The TAA stage is also formed by complementary P‐type and N‐type transconductance stages, each with an R_S_‐C_S_ source‐degeneration bank, which provides up to 6 dB of equalization to compensate for potential on‐chip losses across the entire MPE DPC signal path. Then, the TAA output is followed by TIA‐2, forming a broadband, high‐swing linear voltage‐to‐voltage amplifier (or a Cherry–Hooper amplifier [41]), as TAA and TIA‐2 perform voltage‐to‐current and current‐to‐voltage conversions, respectively. This TAA + TIA architecture possesses two major advantages: First, cascading the TAA and TIA stages significantly improves the AFE output dynamic range and linearity; second, the TIA stage not only increases the bandwidth of the TAA stage due to its low AC input impedance but also provides DC common‐mode feedback, allowing the complementary transconductance in the TAA stage without additional power consumption for setting its output common‐mode voltage.

### 4.3 Mismatch Considerations

Intuitively, there are two major downsides to the proposed MPE sign‐sign DPC accelerator. One is that the differential intensity‐modulation technique seems to require twice as many photonic devices, which can degrade the HS‐DAC bandwidth and consume more on‐chip area in the photonic domain. In reality, performing the sign‐sign DPCs with photonic computing requires twice as many photonic devices anyway, even when using the sign‐magnitude separation approach widely adopted in digital computing systems [34]. The other, more importantly, is device/circuit mismatches due to random process variations, especially in the case of the proposed MPE sign‐sign DPC accelerator, which requires a symmetrical circuit architecture in both the photonic and electronic domains for DPC median‐value cancellation and differential signaling, respectively, as discussed in Section 4.2. Therefore, adequate mismatch corrections are necessary across the entire DPC signal path to maintain the targeted functionality and performance specifications. The analysis and discussion in this subsection primarily focus on photonic device mismatch effects and their correction mechanisms, as this subject in electronic circuits has been well developed and is relatively mature [43].

In the photonic domain of the proposed MPE DPC accelerator shown in Figure 4, the primary sources of mismatches are the random process variations between the photonic devices in the symmetrical pairs MRM_y11_/MRM_y11′_ (or MRM_y12_/MRM_y12′_), MRM_z2p_/MRM_z2n_ (or MRM_z1p_/MRM_z1n_), and PD_p_/PD_n_ (or PD_p′_/PD_n′_). To demonstrate each mismatch effect and its corresponding correction mechanism, the analysis procedure first extracted the standard deviations of the ER mismatches (i.e., *σ*_ERy_ and *σ*_ERz_) within the MRM_y11_/MRM_y11′_ and MRM_z1p_/MRM_z1n_ pairs and the standard deviation of the responsivity mismatch (i.e., *σ*_RPD_) within the PD_p_/PD_n_ pair based on Monte Carlo simulations with the process variation models in the GF45SPCLO PDK. Then, the mismatch of each symmetrical pair was applied individually to analyze the performance impact and the correction effectiveness distinctly. As demonstrated in Figure 5 and summarized in Table 1, the quality of the DPC outcomes is evaluated by the worst nonlinearities (i.e., worst DNL and INL) and the residual median of the analog differential DPC data constellation (i.e., *V*_DPCp_ − *V*_DPCn_) in the electronic domain right before the differential A/D process, as shown in Figure 4, which covers the entire DPC signal path. Note that the signal levels and total DR in Figure 5 and Table 1 are all normalized to (*V*_DPCp_ − *V*_DPCn_)_MAX_ = (y_11_·z_1_ + y_12_·z_2_)_V,MAX_ when y_11,O_ = y_12,O_ = 1.

**TABLE 1:** Individual photonic mismatch effects and corresponding correction mechanisms qualified by the worst DNL, INL, and residual median of the electronic DPC data constellation generated by the MPE sign‐sign DPC accelerator when y_11,O_ = y_12,O_ = 1.

Case	Mismatch condition	Mismatch correction mechanism	DNL in |%| of DR (= 1 LSB of # ENOB)	INL in |%| of DR (= 1 LSB of # ENOB)	Residual median in |%| of DR (= 1 LSB of # ENOB)
1	No mismatch	None	< 2.02% (= 1 LSB of 5.63 bits)	< 2.30% (= 1 LSB of 5.44 bits)	0.92% (= 1 LSB of 6.76 bits)
2	1·*σ*_ERy_ mismatch within the MRM_y11_/MRM_y11′_ pair	None	Eye closed	Eye closed	19.24% (= 1 LSB of 2.38 bits)
		Thermal offset tuning |*V*_th1_ − *V*_th1′_| = Δ*V*_th1_ = 0.1 V	< 2.04% (= 1 LSB of 5.61 bits)	< 2.35% (= 1 LSB of 5.41 bits)	0.94% (= 1 LSB of 6.73 bits)
3	1·*σ*_ERz_ mismatch within the MRM_z2p_/MRM_z2n_ pair	None	Eye closed	Eye closed	84.87% (= 1 LSB of 0.24 bits)
		Thermal offset tuning |*V*_th2_ − *V*_th2′_| = Δ*V*_th2_ = 0.1 V	< 1.91% (= 1 LSB of 5.71 bits)	< 2.27% (= 1 LSB of 5.46 bits)	0.89% (= 1 LSB of 6.81 bits)
4	1·*σ*_RPD_ mismatch within the PD_p_/PD_n_ pair	None	< 2.04% (= 1 LSB of 5.61 bits)	< 2.30% (= 1 LSB of 5.44 bits)	1.99% (= 1 LSB of 5.65 bits)
		Current offset tuning |*I*_OSp_ − *I*_OSn_| = Δ*I*_OS_ = 1.25 μA	< 2.17% (= 1 LSB of 5.52 bits)	< 2.44% (= 1 LSB of 5.36 bits)	0.77% (= 1 LSB of 7.02 bits)

In the case of Figure 5, because y_11,O_ = 0.6 and y_12,O_ = 0.2, the DPC data constellation exhibits only 13 signal levels, whose ideal values are marked by the black lines, out of all 31 possible signal levels (i.e., 5‐bit resolution across all different values of y_11,O_ and y_12,O_) within the normalized DR between −1 and +1. Under the case of no mismatch, the DPC data constellation, as the eye diagram shown in Figure 5a, achieves DNL < 0.63% of DR, INL < 1.00% of DR, and residual median = −0.33% of DR at the computation throughput of 10 Gb/s/DPC (= 5 bits/Sym/DPC × 2 GSym/s) based on the realistic device and circuit models in GF45SPCLO. Once one *σ*_ERy_ of the ER mismatch within the MRM_y11_/MRM_y11′_ pair is applied, the DPC data constellation exhibits a completely erroneous computation outcome (i.e., eye closed) in terms of all signal levels and a residual median as large as −27.88% of DR, as shown in Figure 5b. Although device mismatch is a random variable during chip fabrication, once a chip has been fabricated, this mismatch becomes a static property inherent in the devices, so static thermal tuning with low FSR sensitivity can offer an adequate solution for correcting photonic‐device mismatches at a relatively high resolution. By applying an approximately 100‐mV thermal offset voltage (Δ*V*_th1_) between the MRM_y11_ and MRM_y11′_ thermal control terminals, *V*_th1_ and *V*_th1′_, respectively, one *σ*_ERy_ of the ER mismatch can be well canceled, as the DPC data constellation in Figure 5c achieves DNL < 0.65% of DR, INL < 1.00% of DR, and residual median = −0.40% of DR, which are almost identical to the performance of Figure 5a at the same amount of computation throughput.

To consider the worst‐case nonlinearities, the constant multiplicands are set to y_11,O_ = y_12,O_ = 1 for all four mismatch cases summarized in Table 1 to maximize the DPC signal‐level distribution across the entire normalized DR between −1 and +1. In Table 1, Case 1 is set as the reference with no mismatch; Case 2 includes one *σ*_ERy_ of the ER mismatch within the MRM_y11_/MRM_y11′_ pair with and without the thermal offset tuning mechanism; Case 3 includes one *σ*_ERz_ of the ER mismatch within the MRM_z2p_/MRM_z2n_ pair with and without the thermal offset tuning mechanism; Case 4 includes one *σ*_RPD_ of the responsivity mismatch within the PD_p_/PD_n_ pair with and without the current offset tuning mechanism. Cases 2 and 3 both demonstrate severe performance degradation due to MRM mismatches, which are correctable via their respective thermal offset tuning mechanisms. Case 4 not only indicates that the PD mismatch is correctable by the current offset tuning mechanism but also verifies that the rail‐to‐rail series PD structure primarily affects median‐value cancellation rather than linearity.

## 5 MPE Sign‐Sign MAC Accelerator Measurement

The preliminary test chip of the MPE MAC accelerator was fabricated in the GF45SPCLO process technology, which is capable of monolithically integrating all the required photonic devices and electronic circuits for the MPE accelerator on a single chip [25, 26], as shown in the die photo in Figure 6, without power/area/integration overhead due to heterogeneous interfaces, ESD protection diodes, chip bumps/pads, and interposers/packages between separate photonic and electronic dies.

**FIGURE 6:**
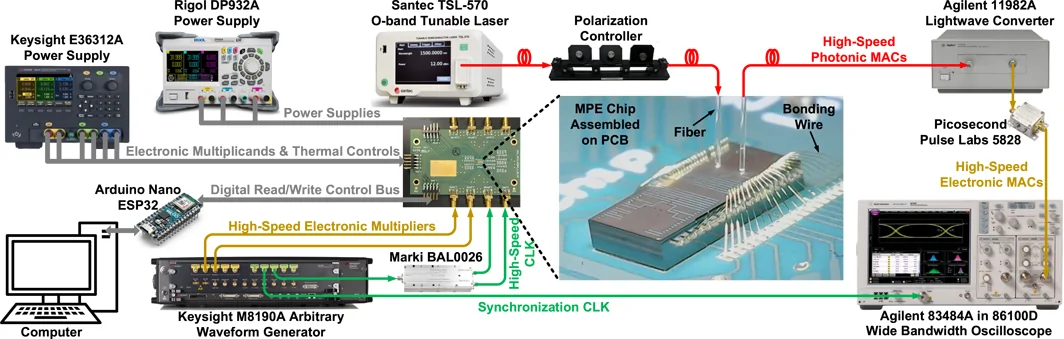
The test bench and die photo of the MPE MAC accelerator test chip.

As shown in the entire test bench for the MPE MAC accelerator in Figure 6, a 0.7‐mW WDM carrier from an external laser source with transverse electric (TE) polarization is injected onto the surface of the MPE test chip via a 15° vertically tilted fiber and an on‐chip grating coupler. Meanwhile, the on‐chip photonic computing outcome can be externally observed via an on‐chip optical power splitter before reaching the on‐chip PD, followed by the other grating coupler and the other 15° vertically tilted fiber, and then measured by off‐chip instruments. The MPE test chip is surface‐mounted on a printed circuit board (PCB), and the electronic high‐speed data/clock, DC bias, power supply, and static control interfaces are all wire‐bonded between the MPE test chip and the PCB, whose high‐speed signal traces are designed as transmission lines with 50‐Ω termination. The ambient temperature of the test bench is air‐conditioned at around 27°C.

Two scenarios of the MAC outcomes, depending on the numbers of bits in the constant multiplicand y_11_ and in the 2‐GSym/s multiplier z_1_, are separately demonstrated. The first scenario is when y_11_ is signed 2.59‐bit data (= 6‐level data constellation distributed over ± 1/5, ± 3/5, and ± 1) and z_1_ is signed 2‐bit data (= 4‐level data constellation distributed over ± 1/3 and ± 1); therefore, the MAC outcome of this y_11_·z_1_ scenario shall exhibit a 16‐level (= 4‐bit) data constellation within a normalized DR = 2, ideally quantized by an LSB = 2/15, as shown in the measured eye diagram in Figure 7a, where it is important to note the following: (i) There are six missing levels (i.e., ± 7/15, ± 11/15, and ± 13/15) because they are not deterministic products of any possible combinations between y_11_ and z_1_ in this scenario. (ii) Because y_11_ is constant data with respect to the high‐speed data z_1_ and the high‐speed MAC outcome y_11_·z_1_, the overall MAC data constellation in Figure 7a is obtained by overlapping three individually measured eye diagrams under the settings of |y_11_| = 1/5, 3/5, and 1, respectively, which are distinguished and marked by three different color depths, as shown in Figure 7a. Therefore, the MAC data constellation does not exhibit any data‐level transitions among different |y_11_| settings (i.e., different color depths), unlike a generic 16‐level eye diagram in random data communications. (iii) The concept of this analog multiplication process in the photonic domain is essentially using y_11_ as a scalar of z_1_, so the signal contents of z_1_, including the data information, noise, and intersymbol interference (ISI), are all scaled by y_11_ accordingly, which is reflected by the larger amount of data‐level dispersions (or spreadings) in Figure 7a when |y_11_| is set to a larger number.

**FIGURE 7:**
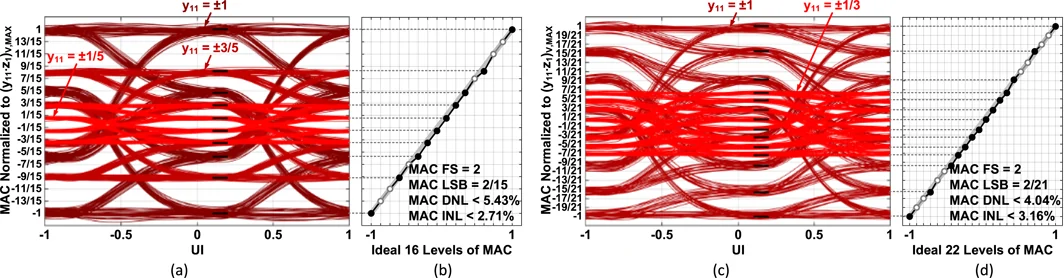
(a) The measured data constellation and (b) the transfer curve of the MPE sign‐sign MAC accelerator, when z_1_ is high‐speed 2‐bit data running at 2 GSym/s with constant y_11_ = ± 1/5, ± 3/5, and ± 1. (c) The measured data constellation and (d) the transfer curve of the MPE sign‐sign MAC accelerator, when z_1_ is high‐speed 3‐bit data running at 2 GSym/s with constant y_11_ = ± 1/3 and ± 1.

By using the center value of each data‐level histogram in the measured eye diagram to form the statistical MAC outcome transfer curve, as shown in Figure 7b, the MPE MAC accelerator in the first scenario can achieve DNL < 5.43% of DR (= 1 LSB of 4.20 bits) and INL < 2.71% of DR (= 1 LSB of 5.20 bits) at a computation throughput of 8 Gb/s/MAC (= 4 bits/Sym/MAC × 2 GSym/s) with an energy efficiency of 2.48 pJ/b/MAC (= 19.8 mW ÷ 8 Gb/s/MAC).

The second scenario is when y_11_ is signed 2‐bit data (= 4‐level data constellation distributed over ± 1/3 and ± 1) and z_1_ is signed 3‐bit data (= 8‐level data constellation distributed over ± 1/7, ± 3/7, ± 5/7, and ± 1); therefore, the MAC outcome of this y_11_·z_1_ scenario shall exhibit a 22‐level (= 4.46 bit) data constellation within a normalized DR = 2, ideally quantized by an LSB = 2/21, as shown in the measured eye diagram in Figure 7c, where it is also important to note the following: (i) There are eight missing levels (i.e., ± 11/21, ± 13/21, ± 17/21, and ± 19/21) because they are not deterministic products of any possible combinations between y_11_ and z_1_ in this scenario. (ii) Because y_11_ is constant data with respect to the high‐speed data z_1_ and the high‐speed MAC outcome y_11_·z_1_, the overall MAC data constellation in Figure 7c is obtained by overlapping two individually measured eye diagrams under the settings of |y_11_| = 1/3 and 1, respectively, which are distinguished and marked by two different color depths, as shown in Figure 7c. Therefore, the MAC data constellation does not exhibit any data‐level transitions among different |y_11_| settings, unlike a generic 22‐level eye diagram in random data communications. (iii) Again, the signal contents of z_1_, including the data information, noise, and ISI, are all scaled by y_11_ accordingly, which is reflected by the larger amount of data‐level dispersions in Figure 7c when |y_11_| is set to a larger number.

By using the center value of each data‐level histogram in the measured eye diagram to form the statistical MAC outcome transfer curve, as shown in Figure 7d, the MPE MAC accelerator in the second scenario can achieve DNL < 4.04% of DR (= 1 LSB of 4.63 bits) and INL < 3.16% of DR (= 1 LSB of 4.98 bits) at a computation throughput of 8.92 Gb/s/MAC (= 4.46 bits/Sym/MAC × 2 GSym/s) with an energy efficiency of 2.22 pJ/b/MAC (= 19.8 mW ÷ 8.92 Gb/s/MAC).

The system‐on‐chip (SoC) power consumption and silicon area of the MPE MAC accelerator are summarized in Table 2 under the case of “*d* = 1,” which covers all the photonic devices in the MRM and PD, the electronic circuits in the HS‐DAC, LP‐DAC, AFE, and ADC, the laser injection power for the WDM carrier, and the heater power for the thermal control and tuning as follows: 0.7‐mW laser injection power per DPC, 5‐mW heater power per DPC, 3‐mW HS‐DAC power per DPC, 0.1‐mW LP‐DAC power per MAC, 6‐mW PD‐and‐AFE power per DPC, 5‐mW ADC power per DPC, 0.005‐mm^2^ MRM area per MAC, 0.0025‐mm^2^ LP‐DAC area per MAC, 0.028‐mm^2^ HS‐DAC area per DPC, 0.006‐mm^2^ PD‐and‐AFE area per DPC, and 0.04‐mm^2^ ADC area per DPC.

**TABLE 2:** The estimated performance of the MPE accelerator and ASIC TPUs for the outlook on upcoming linear‐algebra computation chiplets.

MPE accelerator in GF45SPCLO	# of MAC	Carrier and heater power	Electronic‐circuit power	Total SoC power	Total SoC area	Computation throughput per MAC	Energy efficiency per MAC	Total SoC computation throughput	Total SoC computation density	Total SoC energy efficiency
	*d* ^2^	*P*_P_ = *P*_C_ + *P*_H_ mW	*P*_E_ mW	*P*_SoC_ = *P*_P_ + *P*_E_ mW	*A*_SoC_ mm^2^	Gb/s/MAC	pJ/b/MAC	*d* ^2^·*f*_CLK_ TMAC/s	*d* ^2^·*f*_CLK_/*A*_SoC_ TMAC/s/mm^2^	*P*_SoC_/(*d* ^2^·*f*_CLK_) fJ/MAC
	**1** ^**2**^	**5.7**	**14.1**	**19.8**	**0.0815**	**8.92**	**2.22**	**0.002**	**0.025**	**9900**
	4^2^	22.8	57.6	80.4	0.416	8.92	0.56	0.032	0.077	2513
	16^2^	91.2	249.6	340.8	3.104	8.92	0.15	0.512	0.165	665.6
	64^2^	364.8	1305.6	1670.4	35.456	8.92	0.05	8.192	0.231	203.9
	256^2^	1459.2	10,138	11,597.2	510.46	8.92	0.02	131.072	0.257	88.48

ASIC TPU in CMOS	# of MAC	Total SoC idle power		Total SoC busy power	Total SoC area	Computation throughput per MAC	Energy efficiency per MAC	Total SoC computation throughput	Total SoC computation density	Total SoC energy efficiency
	*d* ^2^	*P*_idle_ mW		*P*_SoC_ mW	*A*_SoC_ mm^2^	Gb/s/MAC	pJ/b/MAC	*d* ^2^·*f*_CLK_ TMAC/s	*d* ^2^·*f*_CLK_/*A*_SoC_ TMAC/s/mm^2^	*P*_SoC_/(*d* ^2^·*f*_CLK_) fJ/MAC
TPUv1 [31]	256^2^	28,000		40,000	331	5.6	0.11	45.88	0.14	871.9
TPUv4 [32]	256^2^	55,000		78,571	400	8.4	0.14	68.81	0.17	1141.8

*Note:* (i) For *d* = 1 in the MPE accelerator section, the performance numbers are based on the measurement results of the MPE test chip described in Section 5. (ii) For *d* > 1 in the MPE accelerator section, the performance numbers are estimated based on the measurement results of *d* = 1. (iii) The busy‐mode power of TPUv4 in the ASIC TPU section is estimated by its idle‐mode power (= 55 W) and the busy‐versus‐idle power ratio of TPUv1 (= 1.43).

## 6 Summary and Future Work

The key performance metrics of linear‐algebra accelerators are computation throughput (TMAC/s), computation density (TMAC/s/mm^2^), and energy efficiency (fJ/MAC) [8, 18, 19, 27, 28]. Based on the measurement results of the MPE MAC accelerator listed in Table 2 for *d* = 1, as well as the breakdowns per DPC (∝ *d*) or per MAC (∝ *d* ^2^) [27, 28] listed at the end of Section 5, the power, area, and performance metrics of an MPE accelerator for *d* = 4, 16, 64, and 256 are estimated and summarized in Table 2, following the projection curves of the SoC power, *P*_SoC_ = (19.7·*d* + 0.1·*d* ^2^) mW, and the SoC area, *A*_SoC_ = (0.074·*d* + 0.0075·*d* ^2^) mm^2^, which indicate significant performance trends of the MPE accelerator in terms of its computation density and energy efficiency as follows: (i) The electronic circuits dominate the SoC power consumption regardless of the number of MACs, *d* ^2^. (ii) However, when *d* increases, the photonic‐device area and computation throughput increase with *d* ^2^, but the electronic‐circuit power and area increase roughly with *d*. (iii) According to (i) and (ii), when *d* increases, each electronic circuit is utilized to serve more photonic devices for a larger computation throughput on average. Essentially, any performance metrics related to *P*_SoC_ and *A*_SoC_ exhibit roughly linear scaling behavior when *d* is small (*d* ≤ 16) due to the domination of the first‐order factors, but quadratic scaling behavior when *d* is large (*d* ≥ 256) due to the domination of *d* ^2^. In sum, for a large scale of *d*, the total power and area of electronic circuits are leveraged by more photonic devices or higher computation throughput. In other words, the MPE computing methodology and MPE accelerator implementation demonstrated in this article would be particularly beneficial for large‐scale linear‐algebra computations in the AI and MIMO applications mentioned earlier.

For reference purposes, the performance metrics of commercial tensor processing units (TPUs) [31, 32] are listed in Table 2 as well because these TPUs implemented by digital application‐specific integrated circuits (ASICs) are also aimed at performing a large‐scale (265^2^ MACs) linear‐algebra computation on a single chip fabricated in advanced CMOS process technologies. In the estimated cases of *d* ≥ 16, the MPE accelerator outperforms the ASIC TPUs in both computation density and energy consumption. In particular, with future work scaling the MPE accelerator to support up to *d* = 256, the MPE accelerator can potentially exhibit about 2× computation throughput, 1.5× computation density, and 13× energy efficiency superiorities over today's TPUs.

Finally, it is important to note that many novel SiPh devices and circuits are still being discovered and engineered for future foundry manufacturing. Therefore, in addition to the power, area, and throughput scalabilities estimated in Table 2, the overall performance metrics of the MPE accelerator can be further enhanced as next‐generation SiPh process technology advances.

## Author Contributions

**Sumilak Chaudhury:** writing – original draft, writing – review and editing, methodology, investigation, validation, formal analysis, software, data curation. **Karl Johnson:** validation, software, data curation. **Nikola Alic:** resources. **Bill Lin:** supervision, funding acquisition, visualization, project administration. **Yeshaiahu Fainman:** supervision, funding acquisition, visualization, project administration, resources, conceptualization, methodology. **Tzu‐Chien Hsueh:** supervision, writing – original draft, writing – review and editing, funding acquisition, visualization, project administration, resources, conceptualization, methodology, investigation, formal analysis.

## Funding

This work was supported in part by the National Natural Science Foundation (Grant Nos. 2023730, 2025752, 2045935, 2217453, and 2410053) and in part by ASML/Cymer Corporation.

## Conflicts of Interest

The authors declare no conflicts of interest.

## Data Availability

The datasets generated and/or analyzed during this study are available from the corresponding author upon reasonable request.
